# Potential Protective Effects of Bioactive Constituents from Chinese Propolis against Acute Oxidative Stress Induced by Hydrogen Peroxide in Cardiac H9c2 Cells

**DOI:** 10.1155/2017/7074147

**Published:** 2017-02-27

**Authors:** Liping Sun, Kai Wang, Xiang Xu, Miaomiao Ge, Yifan Chen, Fuliang Hu

**Affiliations:** ^1^Institute of Apicultural Research, Chinese Academy of Agricultural Sciences, Beijing 100093, China; ^2^National Research Center of Bee Product Processing, Ministry of Agriculture, Beijing 100093, China; ^3^College of Animal Sciences, Zhejiang University, Hangzhou 310058, China

## Abstract

Chinese propolis (CP) is known as a health food but its beneficial effects in protecting cardiomyocytes remain elusive. Here, we investigated the effects of CP and its active compounds on hydrogen peroxide (H_2_O_2_) induced rats cardiomyocytes (H9c2) oxidative injury. Cell viability decreases induced by H_2_O_2_ were mitigated by different CP extracts using various solvents. From these active fractions, six active compounds were separated and identified. Among tested isolated compound, the cytoprotective activities of three caffeates, caffeic acid phenethyl ester (CAPE), benzyl caffeate (BZC), and cinnamyl caffeate (CNC), exerted stronger effects than chrysin, pinobanksin, and 3,4-dimethoxycinnamic acid (DMCA). These three caffeates also increased H9c2 cellular antioxidant potential, decreased intracellular calcium ion ([Ca^2+^]_i_) level, and prevented cell apoptosis. Overall, the cardiovascular protective effects of the CP might be attributed to its caffeates constituents (CAPE, BZC, and CNC) and provide evidence for its usage in complementary and alternative medicine.

## 1. Introduction

Cardiovascular disease (CV) is responsible for 30% of deaths worldwide, surpassed other diseases, and is projected to account for 25 million deaths annually by 2030 [1]. The cost of CV estimated U.S. $863 billion (in 2010) [2]. Myocardial ischemia (MI), commonly known as angina, is one of the major clinical indications of CV and mainly caused by intraluminal coronary thrombosis and ruptured atherosclerotic plaque [3]. Ischemic damages to the cardiac cells are known to be related to reactive oxygen species (ROS) produced during tissue ischemia, which will lead to cardiomyocytes' oxidative stress and further lead to apoptotic cell death [4]. Now one major interesting area is to understand and to prevent cardiac cell death associated with oxidative stress, and several antioxidants have been shown with promising therapeutic effects [5].

Chinese propolis (CP) is an important hive product collected by honeybees (*Apis mellifera*) from buds of plants [6]. CP is widely used as a natural antioxidant and is as a well-known functional food for its biological activities, like anti-inflammatory, antimicrobial, liver detoxifying, and cardioprotective effects [7, 8]. Moreover, the antioxidant basis of CP might attribute to its abundant polyphenolic compounds, mainly flavonids, quercetin, chrysin, kaempferol, pinocembrin, or phenolic acids and its esters, including caffeic acid and CAPE,* p*-coumaric acid [8]. Recently, cardioprotective effect of propolis extract has been investigated both in vitro and in vivo [9]. Although the mechanisms of action beyond these polyphenolic compounds from propolis are not well-defined, it is still convincible that some of their protective effect by propolis can be attributed to direct scavenging properties of these polyphenolic compounds in CP [10].

The study of the material basis and mechanism of natural medicine including propolis always becomes a critical issue for its potential clinical application and modern medicine development [11]. However, mechanisms underlying the cardioprotective effects of CP and its bioactive constituent basis are still lacking. In the present study, we aim to investigate the protective effects of CP against H_2_O_2_ induced rat cardiac H9c2 injury and to explore the potential bioactive compounds of CP.

## 2. Material and Methods

### 2.1. Regents

HPLC grade methanol was purchased from Merck (Darmstadt, Germany) analytical grade solvents were purchased from Beijing Chemical Works (Beijing, China). Quercetin, Fura-2/AM probe, Annexin V-FITC/PI cell apoptosis kit and the standards used in the chromatography analysis were purchased from Sigma (St. Louis, MO, USA). Other chemicals were of analytical grade and purchased from Sangon Biotechnology (Shanghai, China).

### 2.2. Cell Culture and Cell Viability Assay

Rat H9c2 cardiomyocyte cell line was obtained from ATCC (American Type Culture Collection). Cells were cultured using high glucose Dulbecco's modified Eagle's medium with 4.0 mM L-glutamine (Thermo Scientific) supplemented with 1% penicillin/streptomycin (Solarbio, Beijing, China) and 10% fetal bovine serum (Gibco, Carlsbad, CA, USA). Cells were split when a confluence of ~70% was achieved using trypsin-EDTA (Solarbio, Beijing, China) and subcultured at a ratio of 1 : 3. H9c2 cardiomyocytes were seeded at a density of 1.2 × 10^6^ cells/well.

H_2_O_2_ (30%, 3 *μ*L) was diluted by cell culture medium (high glucose DMEM, 3 mL) to obtain 10 mM stock and further diluted to specific working solutions. The H9c2 cells were cultured in DMEM without FBS for 12 h and then treated with various concentrations of H_2_O_2_ for different periods. Cell viability assay was then performed using a CCK-8 kit. Cellular morphology was observed under an inverted microscope.

Cell viability assay was performed using CCK-8 kit (Solarbio, Beijing, China) according to the manufacture's instruction. Then the optical density (OD) at 450 nm for each well was measured by a microplate reader (Synergy HT, BioTek Instruments, Winooski, VT). Cell viability was also confirmed by trypan blue exclusion and microscopy examination during the following experiments.

### 2.3. CP Active Compounds Separation, Determination, and Selection

#### 2.3.1. CP Sample Preparation

CP was collected from Shandong, China, and the botanical origin was poplar (*Populus sp.*). A voucher specimen was deposited at Institute of Apicultural Research, Chinese Academy of Agricultural Sciences, China. The propolis stored at −20°C was grinded into powder with a grinder and extracted by 40% ethanol at 30°C, and then the propolis sample was filtered and concentrated under vacuum using a rotary evaporator. The residues were washed and dried to obtain 40% ethanol upper fraction (40EU), and the supernatants were rotated and dried to obtain 40% propolis extracts (40EL). The 70% ethanol and 95% ethanol were further used to extract from the residues to get 70% ethanol extracted propolis (70E) and 95% ethanol extracted propolis (95E).

#### 2.3.2. Fractional Extraction of CP

Propolis sample was mixed by petroleum ether, ethyl acetate, acetone, and methyl alcohol (the proportion of solid to liquid is 1 : 10). After standing delaminating and evaporation of the solvent, paste-like extraction was obtained. TLC (thin-layer chromatography) condition as followed, chloroform : methanol : formic acid = 8.8 : 0.5 : 1.0. ferric trichloride (2%) as the chromogenic agent.

### 2.4. Separation, Purification, and Determination of Propolis Extracts

#### 2.4.1. Column Chromatography

Column was packed with 200–300 mesh silica gel. After eluting with dichloromethane-acetone system in normal pressure, six components were obtained. Column chromatography was performed by SiliaSphere C18 chromatography column (packed with 300–400 mesh silica gel), eluted with chloroform-methanol, dichloromethane-methanol, petroleum ether-ethyl acetate, and methanol-H_2_O system with a solvent flow rate of 10 mL/min in medium-pressure.

#### 2.4.2. High-Performance Liquid Chromatography (HPLC) Analysis

HPLC analyses were carried out on an Agilent HPLC system. The separation was performed on Agilent C18 column (250 mm × 4.6 mm, 5 *μ*m) with a flow rate of 1.0 mL/min at 40°C, eluted with methanol-H_2_O system. The preparative HPLC was equipped with an Agilent SB C18 column (150 mm × 21.2 mm, 5 *μ*m) with a flow rate of 20 mL/min at 40°C.

#### 2.4.3. LC-MS Determination

An Agilent SB C18 column (2.1 mm × 50 mm, 1.8 *μ*m) was used for the separation at a flow rate of 0.2 mL/min. The mobile phase comprised aqueous 60% methanol. The column was maintained at 40°C. Mass spectrometer operated in negative and positive full-scan mode in the range 100–1000 Da. The capillary voltage set to 4.0 kV and the desolvation temperature was 350°C. The cone gas flow was set at 6 L/h, while the desolvation gas flow was set to 9 L/h, and the collision energy at 20 V.

### 2.5. Cellular Antioxidant Activity Determination

#### 2.5.1. Measurement of Cellular SOD Activity

The H9c2 cells were seeded into 24-well plates at a density of 1 × 10^5^ cells/mL. Then the DMEM of propolis treated group was removed and 1% Triton × 100 was added into cells and incubated for 30 min. After this incubation, cells were collected by centrifugation at 2500 rpm for 10 min. The supernatants separated were used 100 ul for measurement of SOD activities according to the manufacturer's instructions (Jiancheng Bioengineering Institute, Nanjing, China).

#### 2.5.2. Measurement of MDA Activity

Cell culture and treatment methods were as described. We collected 150 *μ*L supernatants of cell lysate for MDA levels measurement according to the manufacturer's instructions (Jiancheng Bioengineering Institute).

#### 2.5.3. Measurement of GSH-Px Activity

Supernatants (200 *μ*L) were used for measurement of* GSH-Px* content according to the manufacturer's instructions (Jiancheng Bioengineering Institute).

### 2.6. Determination of Intracellular Calcium Ion ([Ca^2+^]_i_)

H9c2 cells were digested and seeded into culture plate (10^5^ cells/mL) at 37°C in a 5% CO_2_ atmosphere. The cell medium was discarded and washed cells with HBSS buffer solution 3 times, then added Fura-2/AM and incubated 45 min at 37°C in the dark. After the incubation, the cells were washed 2-3 times with HBSS solution and then 2 mL EBSS buffer solution was loaded. Fura-2 fluorescence was excited alternately at 340 and 380 nm and the 340/380 ratio was obtained. Values for *R*min, *F* 380 max, *F* 380 min, *R*max and *Kd* were obtained using the Fura-2 Calcium Imaging Calibration Kit (Molecular Probes).

### 2.7. Cell Apoptosis Analysis Using Flow Cytometry

Cardiomyocytes were labeled with Annexin V-FITC and PI, and apoptosis rate was measured by flow cytometry using a Cell Llab Quanta™ SC flow cytometer (Beckman Coulter Inc., Miami, FL). H9c2 cells were digested and seeded into culture plate to a density of 5 × 10^5^ cells/mL at 37°C, 5% CO_2_ before the experiment. Cells were then centrifuged at 1000 rpm 5 min and washed 2 times with PBS and 500 *μ*L of conjugation buffer was added to resuspend the cells. Then 5 *μ*L Annexin V-FITC and 10 *μ*L PI were loaded and reacted for 10 min before FCM analysis.

### 2.8. Statistical Analysis

All experiments were performed in triplicate, and each experiment was repeated at least three times. All values are presented as mean ± SD. The data were analyzed by one-way analysis of variance followed by post hoc Dunnett's *t*-test for multiple comparisons. Values of *P* < 0.05 were considered to be statistically significant.

## 3. Results and Discussion

### 3.1. Oxidative Damage Model Establishment in H9c2 Cardiomyocytes

Myocardial damage is largely due to the generation of ROS. ROS-induced effects of ischemia–reperfusion and myocardial dysfunction could be alleviated by treating the tissue with antioxidants or blocking signaling-related ROS generation. Based on this concept, several practices have been performed to evaluate the effects of antioxidants, including propolis, on myocardial injuries in animals and patients [12, 13].

**Figure 1:**
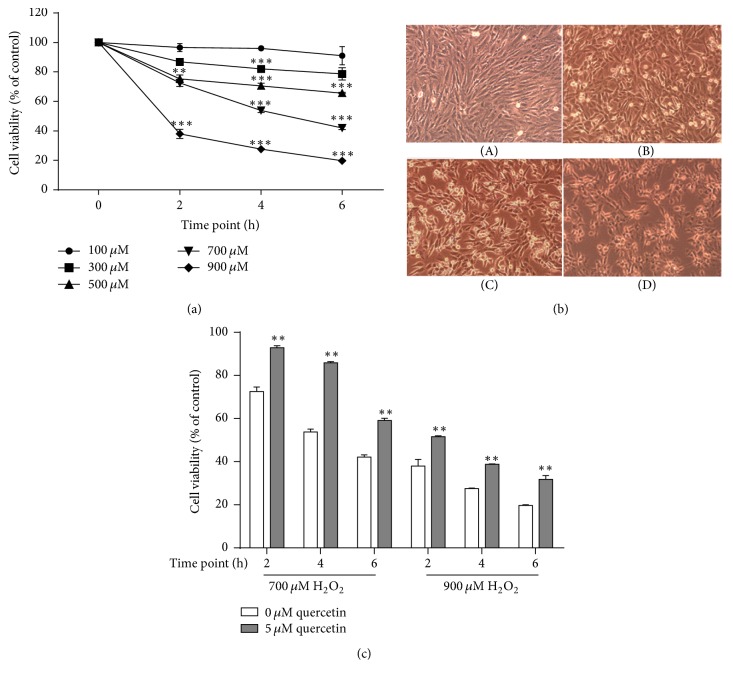
Oxidative damage model establishment in H9c2 cardiomyocytes. (a) H9c2 cells were treated with various concentrations of H_2_O_2_ (100 *μ*M, 300 *μ*M, 500 *μ*M, 700 *μ*M, and 900 *μ*M) for different time periods (2, 4, and 6 h). (b) H9c2 cell morphology from 700 *μ*M H_2_O_2_ stimulation for 0 h (A), 2 h (B), 4 h (C), and 6 h (D). (c) Comparisons of cell survival rates between quercetin treatment group and oxidative injury group. H9c2 cardiomyocytes were pretreated with 5 *μ*M quercetin or not for 12 h and then challenged with 700 or 900 *μ*M H_2_O_2_ for 2 h, 4 h, and 6 h. ^*∗∗*^*P* < 0.01 and ^*∗∗∗*^*P* < 0.001 compared with H_2_O_2_ controls.

We challenged H9c2 cardiomyocytes with various concentrations of H_2_O_2_ (0–900 mM) for different time periods (0 to 6 h) to induce in vitro oxidative damage. As shown in Figure 1(a), H9c2 cell viability decreased significantly in a time- and dose-dependent manner after H_2_O_2_ treatment. Six hours after H_2_O_2_ challenge, more than 50% cell viability losses were observed in 700 and 900 mM H_2_O_2_ H9c2 cells (*P* < 0.01 compared with control cells). In parallel, damaged cell morphology was observed using an inverted microscope, shown as broken cellular membranes, swelling, and vacuole degeneration in 700 mM H_2_O_2_ treated H9c2 cells (Figure 1(b)), which were quite similar to several previous studies using H9c2 cells [14].

Propolis has abundant polyphenolic constituents, like flavonoids and phenolic acids [15]. These constituents are known with good antioxidant, iron-chelating, and carbonyl reductase-inhibitory effects, which act as new protective compounds against cardiotoxicity [16–18]. It has been reported that quercetin, an important flavonoid abundantly found in fruits, vegetables, wine, and tea (also found in CP [10, 19]), exerts protective effects against H_2_O_2_ cardiotoxicity in H9c2 cardiomyocytes [20, 21]. We used 5 *μ*M quercetin as a positive control in this study, which rescued cell viability from 53.9% to 86.0% in 700 mM H_2_O_2_ treated H9c2 cells for 4 h (Figure 1(c)), whereas 900 mM H_2_O_2_ insult with 5 *μ*M quercetin pretreatment can only rescue H9c2 cell viability to 60%, suggesting that oxidative damage induced by this concentration H_2_O_2_ was irreversible. Therefore, in our system, 700 *μ*M H_2_O_2_ challenge for 4 h was chosen in the subsequent experiments as an oxidative damage model.

### 3.2. CP Extracts and Its Fractions Inhibit H_2_O_2_-Induced Cell Death in H9c2 Cardiomyocytes

**Figure 2:**
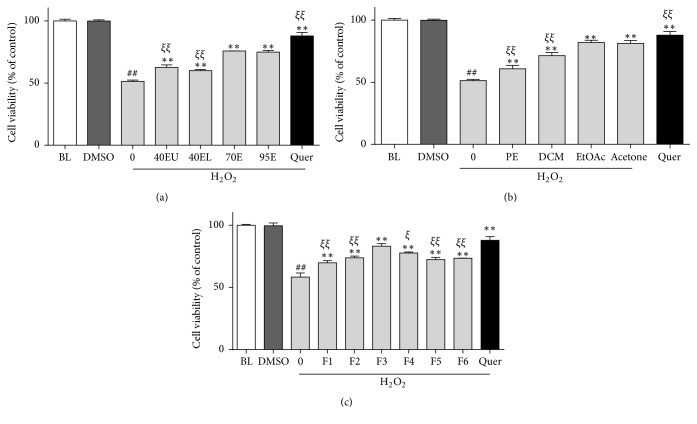
(a) Effects of different alcoholic extracts of CP on H9c2 cardiomyocytes cell viability decreases induced by H_2_O_2_ (700 *μ*M for 6 h). H9c2 cardiomyocytes were pretreated with 20 *μ*g/mL alcoholic extracts of propolis, that is, 70% ethanol fraction (70E), 95% ethanol fraction (95E), 40% ethanol upper fraction (40EU), and 40% ethanol lower fraction (40EL). Quercetin (5 *μ*M) served as a positive control. ^*∗∗*^*P* < 0.01 versus oxidative injury group, ^##^*P* < 0.01 versus control group, and ^*ξξ*^*P* < 0.01 versus 70E group. (b) Effects of different fractions from CP using different solvents on H9c2 cardiomyocytes cell viability decreases induced by H_2_O_2_ (700 *μ*M for 6 h), that is, petroleum ether (PE), dichloromethane (DCM), ethyl acetate (EtOAc), and acetone fractions. Quercetin (5 *μ*M) served as a positive control. ^*∗∗*^*P* < 0.01 versus oxidative injury group, ^##^*P* < 0.01 versus control group, and ^*ξξ*^*P* < 0.01 versus EtOAc group. (c) Effects of different subfractions (fractions 1 to 7) from CP EtOAc/acetone fraction on H9c2 cardiomyocytes cell viability decreases induced by H_2_O_2_ (700 *μ*M for 6 h). ^*∗∗*^*P* < 0.01 versus oxidative injury group, ^##^*P* < 0.01 versus control group, and ^*ξξ*^*P* < 0.01, ^*ξ*^*P* < 0.05 versus fraction 3 group.

It has been known that different solvents will affect yield of bioactive constituents, like flavonoids or phenolic acids [22]. To find active cardioprotective fractions from CP, different ethanol CP extracts were screened in H_2_O_2_ challenged H9c2 cells. As shown in Figure 2(a), among all CP fractions tested, 70% ethanol fraction (70E) showed strongest protective effects against H_2_O_2_ challenge (with a cell viability of 51.3 ± 1.0%), in which the cell viability significantly increased to 75.8 ± 0.2% (*P* < 0.01). Nevertheless, both 40% ethanol upper fraction and lower fraction (40EU and 40EL) showed less potent protective effects against H9c2 cell viability decreases, which were significant from 70E fraction. Since the protective effects of 90% ethanol fraction (90E) were quite similar to 70E fraction, we merged them for the following fractionation.

Further, merged 70E and 90E fractions were sequentially fractionated into five subextracts explicitly, namely, petroleum ether (PE), dichloromethane (DCM), ethyl acetate (EtOAc), and acetone (thin layer chromatography, TLC, profile of these fractions was shown in Supplemental Figure 1 in Supplementary Material available online at https://doi.org/10.1155/2017/7074147). As shown in Figure 2(b), EtOAc subfraction and acetone subfraction showed most effective protective effects and kept for next fractionation.

Subsequently, combined EtOAc and acetone subfractions were separated over a silica gel column and six subfractions were obtained (TLC profile was shown in Supplemental Figure 2). As shown in Figure 2(c), the cytoprotective effect by fraction 3 was the strongest among all tested fractions, which was comparable to the quercetin positive control. Based on these results, we chosen this fraction for the active compound isolation and structure elucidation.

### 3.3. Active Compounds Isolation and Characterization of CP Extracts Responsible for Its Cardioprotective Activities

**Figure 3:**
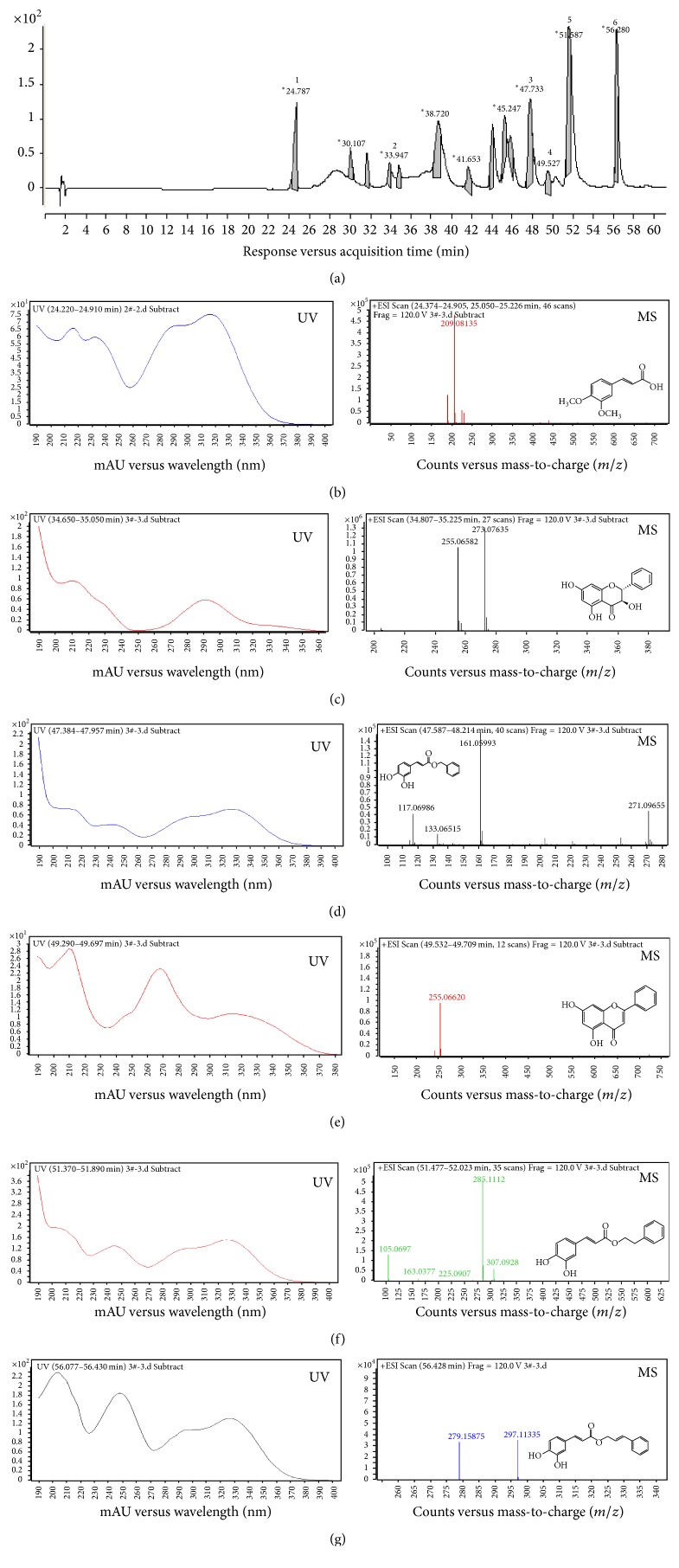
Active compounds isolation and characterization of CP extracts responsible for its cardioprotective activities. (a) HPLC chromatogram of CP fraction 3. Peaks present are as follows: (1) 3,4-dimethoxycinnamic acid (DMCA); (2) pinobanksin; (3) benzyl caffeate (BZC); (4) chrysin; (5) caffeic acid phenethyl ester (CAPE); and (6) cinnamyl caffeate (CNC). Their UV (left) and MS profiles (left) are shown individually as (b) DMCA; (c) pinobanksin; (d) BZC; (e) chrysin; (f) CAPE; (g) CNC.

Fraction 3 from EtOAc and acetone sub-fraction was repeatedly separated by silica gel column chromatography, purified by reverse phase HPLC (Figure 3(a)), six known compounds, which is (1) 3,4-dimethoxycinnamic acid (DMCA) [23], (2) pinobanksin [24], (3) benzyl caffeate (BZC) [25], (4) chrysin [23], (5) caffeic acid phenethyl ester (CAPE) [23, 26] and (6) cinnamyl caffeate (CNC) [23, 27].

Next, we examined different concentrations of these isolated active compounds on H_2_O_2_ induced H9c2 cell viability decreases. Compared to oxidative damage control group, 10 *μ*M of tested compounds showed significant protective effects (*P* < 0.01). These results provided further evidence which previously stated that chrysin can alleviate acute cardiotoxicity in rats [28].

Caffeic acid esters, major active compounds widely found in propolis, were reported to have a wide range of biological effects, which were also quite similar to propolis, like antitumour, antioxidant, and anti-inflammatory activities [13]. We noticed that three major caffeate derivatives, BZC, CAPE, and CNC, exerted stronger protective effects than the remaining three polyphenolic acids (DMCA, chrysin, and pinobanksin) in damaged H9c2 cells (Figure 3(b)). These caffeate derivatives were further investigated regarding their cardiac cell protective mechanisms.

### 3.4. Caffeate Derivatives in CP Increased Cellular Antioxidant Activities in H9c2 Cardiomyocytes

**Figure 4:**
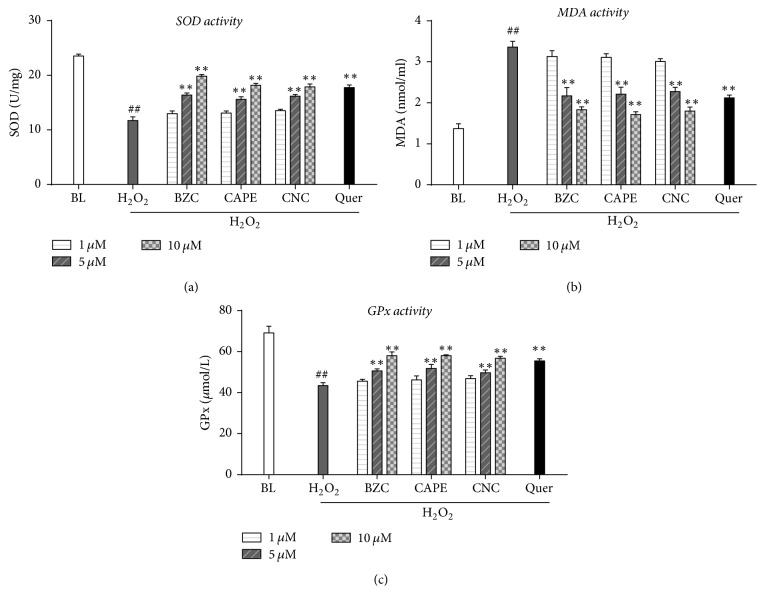
Caffeate derivatives in CP increased cellular antioxidant activities in H9c2 cardiomyocytes. Effects of CP bioactive compounds (BZC, CAPE, and CNC) on cellular SOD (a), MDA, and GPx activities in H_2_O_2_ induced injured H9c2 cardiomyocytes. CP bioactive compounds (1, 5, and 10 *μ*M) were pretreated for 12 h before 6 h H_2_O_2_ (700 *μ*M) insult. ^*∗∗*^*P* < 0.01 versus oxidative injury group and ^##^*P* < 0.01 versus control group.

Oxidative stress was considered as a major challenge during the cardiac ischemic damages, which can be reflected by measurement of the products of free-radical attack on biological substrates (MDA) as well as intracellular and extracellular anti-oxidant capacity (SOD and GSH-Px). MDA is recognized as an indirect oxidative stress marker of cellular injury due to itself is one of the end products of lipid peroxidation [29]. SOD and GSH-Px are known as main endogenous antioxidant enzymes which can neutralize free radicals and protect cells from ROS insults [30]. In our hands, we found that H9c2 cells exposed to H_2_O_2_ showed a significant increase in MDA levels (Figure 4(a)), which was ameliorated by pretreatment with 5 and 10 *μ*M caffeate derivatives (BZC, CAPE, and CNC). In addition, activity of endogenous antioxidative enzymes, SOD (Figure 4(b)) and GSH-Px (Figure 4(c)), was significantly decreased by H_2_O_2_ exposure. However, pretreatments of BZC, CAPE and CNC effectively mount up SOD and GSH-Px dose-dependently, suggesting that these caffeate derivatives isolated from CP might reduce oxidative damage by enhancing the endogenous antioxidant capacity in H9c2 cardiomyocytes.

### 3.5. Caffeate Derivatives in CP Decreased the H_2_O_2_ Induced Elevation of [Ca^2+^]_i_ and Inhibited Cell Apoptosis in H9C2 Cardiomyocytes

**Figure 5:**
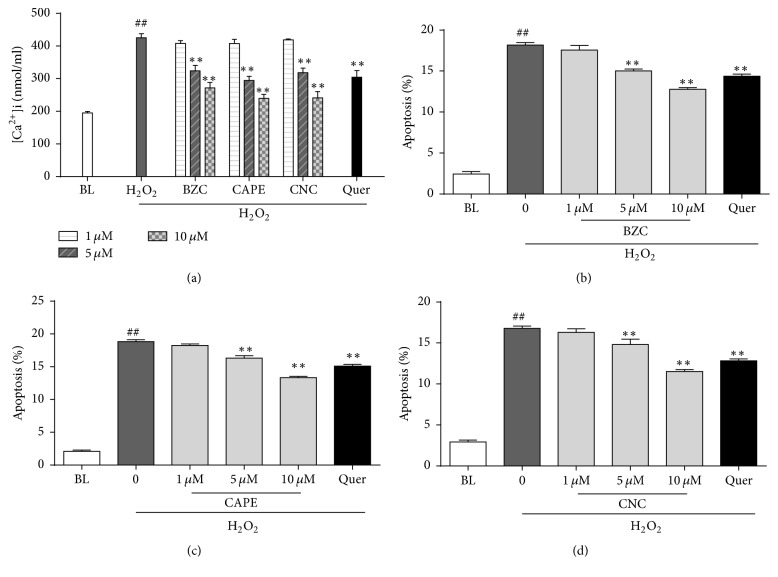
Caffeate derivatives in CP decreased the H_2_O_2_ induced elevation of [Ca^2+^]_i_ and inhibited cell apoptosis in H9c2 cardiomyocytes. (a) Effects of CP bioactive compounds (BZC, CAPE, and CNC) on intracellular [Ca^2+^]_i_ induced by H_2_O_2_. H9c2 cells were pretreated with BZC, CAPE, and CNC for 12 h and then stimulated with 6 h H_2_O_2_ (700 *μ*M). [Ca^2+^]_i_ was measured as described in materials and methods. (b–d) Effects of CP bioactive compounds on cell apoptosis induced by H_2_O_2_. Various concentrations (1, 5, and 10 *μ*M) of BZC (b), CAPE (c), and CNC (d) were added into cell medium for 12 h and then stimulated with 6 h H_2_O_2_ (700 *μ*M). Cell apoptotis were analysed using FCM as described in Materials and Methods. ^*∗∗*^*P* < 0.01 versus oxidative injury group and ^##^*P* < 0.01 versus control group.

Calcium is frequently played in the oxidative stress induced cellular injury [31, 32]. In order to further investigate the protective effects by CP active constituents, [Ca^2+^]_i_ were determined in H9c2 cells (Figure 5(a)). All of three caffeate derivatives (5 and 10 *μ*M) pretreatment significantly brought back myocardial ionizable calcium to near positive quercetin control. These data are consistent with a previous study in human endothelial cells (HUVEC) that cytosolic [Ca^2+^]_i_ were also increased by CAPE [33]. Also, these results provided novel evidence for the other two caffeates, which have promising potential in regulating cellular calcium homeostasis [34].

Myocardial ischemic damages can be affected by apoptosis through at least three potential mechanisms: (1) reducing myocardial cell numbers, which will directly decrease the heart pumping function; (2) damaging the heart's conduction function; and (3) initiating myocardial remodeling and inducing other cardiac pathological changes [29, 35]. For quantitative analyses of myocardial apoptosis, flow cytometry (FCM) with Annexin V-FITC and PI staining was used to detect H9c2 cellular apoptosis. Compared to H_2_O_2_ damage group (16.8%–18.8% apoptotic cell rates), significant lower apoptotic cells were observed in caffeate derivatives pretreated cells (5 and 10 *μ*M). Based on these results, we demonstrated that caffeates from CP could reduce the myocardial injury by inhibiting H_2_O_2_-induced cell apoptosis and restored cytosolic [Ca^2+^]_i_ pool.

## 4. Conclusion

In summary, our study provides an important basis for the use of Chinese propolis for the prevention and treatment of cardiovascular diseases. The crude Chinese propolis extract as well as its isolated compounds caffeate derivatives (CAPE, BZC, and CNC) could mostly possibly be useful for the development of new antimyocardial ischemia drugs, depending on their in vitro activity. However, further in vivo pharmacological and toxicity studies are necessary for its potential clinical usages.

## Supplementary Material

Thin Layer Chromatography (TLC) maps of different extracts (S1) and combined fraction extracts of Chinese propolis.
