# Supplementary material for: Potential Protective Effects of Bioactive Constituents from Chinese Propolis against Acute Oxidative Stress Induced by Hydrogen Peroxide in Cardiac H9c2 Cells
Source: Evid Based Complement Alternat Med. 2017 Feb 27;2017:7074147. doi: 10.1155/2017/7074147 (PMC5350327; doi:10.1155/2017/7074147)
Supplement: Supplementary file 1 — Thin Layer Chromatography (TLC) maps of different extracts (S1) and combined fraction extracts of Chinese propolis. [file 7074147.f1.docx]

Supplemental Materials


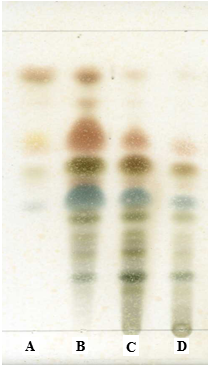


**Rf=0.35~0.86**

**Rf=0.24~0.67**

**Supplemental Figure 1 Thin Layer Chromatography (TLC) maps of different extracts of Chinese propolis**

Fractions represented as (A) petroleum ether (PE) fraction, (B) dichloromethane (DCM) fraction, (C) ethyl acetate (EtOAc) fraction and (D) acetone fraction


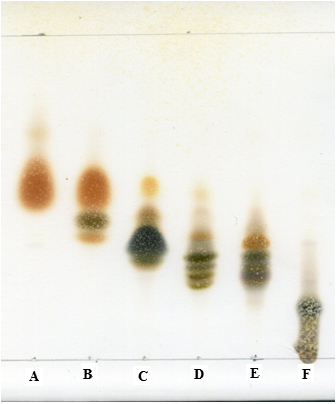


**Rf=0.26~0.56**

**Supplemental Fig.2 TLC map of compositions from Chinese propolis**
